# Supplementary material for: Novel anti-malarial drug strategies to prevent artemisinin partner drug resistance: A model-based analysis
Source: PLoS Comput Biol. 2021 Mar 25;17(3):e1008850. doi: 10.1371/journal.pcbi.1008850 (PMC8023453; doi:10.1371/journal.pcbi.1008850)
Supplement: S1 Appendix — (DOCX) [file pcbi.1008850.s001.docx]

**SUPPORTING INFORMATION: APPENDIX 1**

**Novel anti-malarial drug strategies to prevent artemisinin partner drug resistance: a model-based analysis**

Amber Kunkel, Michael White, and Patrice Piola

**Outline**

1. Parameter tables and descriptions
2. Model fits
3. Extended duration of chemoprophylaxis

**1. Parameter tables and descriptions**

The following variables were held fixed in the model:

| Parameter | Description | Value | Reference |
| --- | --- | --- | --- |
| Fixed parameters – Same for East and West | | | |
| b | Infectiousness of asymptomatic humans (relative to symptomatic) | 1/10 | [1–3]  Assumed most asymptomatic carriers in GMS have submicroscopic infections ([4]) |
| w | Rate of waning immunity | 1/365 days^-1^ | Similar to other models [5,6] |
| w_t | Rate of waning protection by artemisinin partner drug | 1/20 days^-1^ | [7–9] assume average 20 days after leaving I compartment |
| r_a | Clearance rate of asymptomatic infections | 1/60 days^-1^ | [5,10,11] |
| r_s_0 | Clearance rate of symptomatic infectious, assuming no relapse (i.e. treatment is received and is effective) | 1/10 days^-1^ | [5,12,13] |
| s_a | Proportion of individuals receiving non-first-line treatment who do not relapse | 0.5 | 0.75 is the approximate percentage that recrudesce following artemisnin monotherapy, previously widely available in Cambodia [14–19]; reduced to account for poor drug quality, insufficient doses, ART resistance, or individuals receving no treatment |
| s_m | Proportion of individuals with multiple copies of pfmdr1 who do not relapse when treated with ASMQ | 0.75 | comparisons of resistance data (see below); and personal communications with Benoit Witkowski |
| s_p | Proportion of individuals with multiple copies of pfpm2 who do not relapse when treated with DP | 0.5 | comparisons of resistance data (see below); and personal communications with Benoit Witkowski |
| (N/A) | Proportion of treatment failures that move from symptomatic to asymptomatic | 0.1 | Assumed (given oversimplification of model); high values are not able to replicate observed resistance trends |
| mu_0 | Human rate of exiting the population at risk | 1/(365*30) days^-1^ | Malaria risk is greatest among young men who engage in forest activities [20,21]; here, we assume concentrated in ages 15-45 |
| mu_m | Mosquito mortality rate | 1/8 | average life expectancy 8 days [5,22] |
| p_h | Human rate of progression from latency to infectiousness | 1/10 days^-1^ | average latent period 10 days [5] |
| p_m | Mosquito rate of progression from latency to infectiousness | 1/14 days^-1^ | average latent period 14 days [5,23,24] |
| a_0 | Proportion of infectious individuals who acquire resistance upon treatment | 1/1,000,000 | Selected to allow constant low levels of resistance introduction [25] |
| mosq_a | Human biting rate | 1/3 | [5,22] |
| mosq_b | Probability a bite by an infected mosquito leads to infection in a susceptible human | 1/2 | [22,26] |
| mosq_c | Probability a bite of an infected human leads to infection in a susceptible mosquito | 1/2 | [5,22] |
| (NA) | Probability that superinfection of an asymptomatically infected individual, when it occurs, leads to a change in the dominant infecting strain | 0.5 | Intermediate superinfection value |
| rc (recombination coefficient) | Probability that (human) superinfection leads to a specific recombination prior to next human infection | 0.05 | Arbitrarily selected to allow recombination to occur at low levels |
| Fixed parameters - West | | | |
| (NA) | Human population denominator for:  a) Prevalence surveys  b) Case counts from Maude 2014 | 2,700,000 | [27–29]; details of calculations given in text below |
| mdr_2000 | Proportion of malaria parasites with multiple copies of pfmdr1 in 2000 | 15% | [30,31] |
| ppq_init | Proportion of malaria parasites with multiple copies of pfpm2 in 2008 (date of DHA-PPQ introduction) | 1% | [32] |
| Fixed parameters - East | | | |
| (NA) | Human population denominator for:  a) Prevalence surveys  b) Case counts from Maude 2014 | 560,000 | [27–29]; details of calculations given in text below |
| mdr_2000 | Proportion of malaria parasites with multiple copies of pfmdr1 in 2010. Note this is fixed to 0 pre-2010 given the apparent low proportion in 2010 and lack of data prior to that | 10% | [33–35] |
| ppq_init | Proportion of malaria parasites with multiple copies of pfpm2 in 2010 (date of DHA-PPQ introduction) | 1% | [32,34] |

Note on calculation of population denominators:

The 2013 malaria survey used sampling frame of 3.7 million people, but this is not reported by domain (East vs West) [28].

Taking the rural population from the Western provinces included in [29] gives a population denominator of about 560,000 in the East:

Rural population sizes [27]:

Kratie: 282,088

Mondulkiri: 55,809

Rattanakiri: 130,585

Steung Treng: 95,550

Total Northeast: 564,032

In defining the population truly at risk below we focus just on those who spent at least 1 night in the forest in the last 6 months. Extrapolating these percentages for 2010 and 2013 backwards [28,36], we set the median of the prior distribution for the size of the population at risk as if 15% of people in 2004 would meet this definition. However, given the uncertainty in the definition of “at risk” and the necessary extrapolation, this parameter is allowed to vary based on the data (see table of fitted variables below).

Similarly, taking the rural population from the Western provinces included in [29] gives 2.7 million in West:

Rural population sizes [27]:

Banteay Meanchey: 494,462

Battambang: 844,345

Oddar Meanchey: 166,609

Pailin: 54,800

Pursat: 371,524

Siem Reap: 723,466

Total West: 2,655,206

We take 2,700,000 as the population denominator for the West. Under the same reasoning as above, we set the median of the prior distribution for people at risk in the West to about 400,000 people in 2004.

Note on superinfection and recombination:

Multi-strain infections are not tracked in this model, rather, only a dominant infecting strain is considered. Superinfection is allowed to occur during asymptomatic infection and assumed to lead to a change in the dominant infecting strain 50% of the time. To allow for recombination while maintaining simplicity of the model, recombination events that would occur after a blood meal are instead assumed to do so at the time of superinfection. Recombination is allowed to lead to changes in resistance patterns as follows:

^n^I + ^mp^I -> (0.5-rc)* ^n^I + (0.5-rc)* ^mp^I + rc* ^m^I + rc* ^p^I

^m^I + ^p^I -> (0.5-rc)* ^m^I + (0.5-rc)* ^p^I + rc* ^n^I + rc* ^mp^I

Where I indicates infectious individuals, rc is the recombination coefficient, superscript n denotes no partner drug resistance, superscript m denotes genotypic MQ resistance, superscript p denotes genotypic PPQ resistance, and superscript mp denotes genotypic resistance to both MQ and PPQ.

The following variables were fixed but time dependent:

| Parameter | Description | Value | Reference |
| --- | --- | --- | --- |
| Time dependent parameters – same for East and West | | | |
| app | Proportion of symptomatic individuals receiving first line treatment | 0.3 in 2004  0.8 in 2010  Linear increase in between | [17,19,36,37] |
| (NA) | Year treatment became standardized | 2000 (Before this, I assume all get “non-first-line treatment” and do not track resistance) | [38] |
| (NA) | Years of declining transmission attributable to decrease in man-biting rate (maximum: mosq_a = 1/3, minimum: mosq_a*beta_min_m, a fitted parameter) | 2008 to 2012  Assume linear decrease in between | large increase in ITN coverage from 2008-2012 [29] |
| (NA) | Years of declining population at risk | 2004 to 2018  Assume exponential decrease in between to simplify encoding as increased rate of population exit | apparently limited deforestation in early 2000s compared to after [39]  Decline in “people who go to the forest” between 2004 and 2007 [17,18] and “percent who stay in the forest overnight” between 2010 and 2013 [28,36] |
| (NA) | Relative size of population at risk in 2013 vs 2010 | 0.7 | % spending overnight in forest from surveys: 2010 (7.5 %); 2013 (5%) [28,36]  % who go to forest: 20% (2004); 15% (2007) [17,18]  The percentages from 2010 and 2013 are similar for East and West; I assume this also holds pre-2010 |
| Time dependent parameters - West | | | |
| (NA) | Years ASMQ was first line treatment | 2000 to 2008  2017 to present | [38][40]; and personal observation and discussions regarding timing of return to ASMQ |
| (NA) | Years DP was first line treatment | 2008 to 2017 | See citations for ASMQ |
| Time dependent parameters - East | | | |
| (NA) | Years ASMQ was first line treatment | 2000 to 20010  2018 to present | [40,41]; and personal observation and discussions regarding timing of return to ASMQ |
| (NA) | Years DP was first line treatment | 2010 to 2018 | See citations for ASMQ |

Prior distributions of fitted variables

Variables were fit separately for Eastern and Western Cambodia

| Parameter | Description | Distribution | Median | Lower end of 95% interval | Upper end of 95% interval | Reference |
| --- | --- | --- | --- | --- | --- | --- |
| Same Priors but Fitted Separately for East and West | | | | | | |
| mosq_m | Ratio of number of mosquitoes to humans | Lognormal(mu=1, sigma=1)  days^-1^ | 0.61 | 0.38 | 19.3 | [22] Intended to allow wide range |
| beta_min_m | mosq_a*beta_min_m is the man biting rate after decline ends, at median of seasonal amplitude | Logitnormal(mu=-0.2, sigma=1.02) | 0.45 | 0.10 | 0.86 | Intended to allow wide range |
| a | Seasonal amplitude parameter | Logitnormal(mu=0, sigma=0.7) | 1 | 0.2 | 0.8 | Intended to allow wide range |
| p | Probability a symptomatic case is notified | Logitnormal(mu= -1.55, sigma=0.3) | 0.18 | 0.11 | 0.28 | [42] |
| f_m | Relative fitness of strains with multiple copies of pfmdr1. Arbitrarily assumed to apply to human -> mosquito transmission only | Logitnormal(mu=3.4, sigma=0.6) | 0.97 | 0.90 | 0.99 | Assume fitness costs are relatively low [43,44] |
| f_p | Relative fitness of strains with multiple copies of pfpm2. Arbitrarily assumed to apply to human -> mosquito transmission only | Logitnormal(mu=3.4, sigma=0.6) | 0.97 | 0.90 | 0.99 | As for f_m |
| West-specific priors | | | | | | |
| N_init | Initial size of population at risk (forest-goers and people living in forested villages with active malaria transmission) | lognormal(mu=12.9, sigma=0.4) | 400 thousand | 183 thousand | 877 thousand | See description under fixed parameter table |
| East-specific priors | | | | | | |
| N_init | Initial size of population at risk (forest-goers and people living in forested villages with active malaria transmission) | lognormal(mu= 11.35, sigma=0.4) | 85 thousand | 39 thousand | 186 thousand | See description under fixed parameter table |

Prior vs Posterior Distributions

|  |  | Prior | | | Posterior | | |
| --- | --- | --- | --- | --- | --- | --- | --- |
|  | Parameter | Median | Lower end of 95% interval | Upper end of 95% interval | Median | Lower end of 95% interval | Upper end of 95% interval |
| East | mosq_m | 0.61 | 0.38 | 19.3 | 4.16 | 3.74 | 4.57 |
| West |  |  |  |  | 2.39 | 2.28 | 2.55 |
| East | beta_min_m | 0.45 | 0.10 | 0.86 | 0.93 | 0.90 | 0.96 |
| West |  |  |  |  | 0.96 | 0.93 | 0.99 |
| East | a | 0.5 | 0.20 | 0.80 | 0.63 | 0.56 | 0.69 |
| West |  |  |  |  | 0.31 | 0.28 | 0.33 |
| East | p | 0.18 | 0.11 | 0.28 | 0.26 | 0.22 | 0.32 |
| West |  |  |  |  | 0.15 | 0.14 | 0.17 |
| East | f_m | 0.97 | 0.90 | 0.99 | 0.97 | 0.96 | 0.99 |
| West |  |  |  |  | 0.96 | 0.96 | 0.97 |
| East | f_p | 0.97 | 0.90 | 0.99 | 0.91 | 0.90 | 0.92 |
| West |  |  |  |  | 0.88 | 0.86 | 0.89 |
| East | N_init | 85k | 39k | 186k | 133k | 115k | 150k |
| West |  | 400k | 183k | 877k | 341k | 301k | 377k |

**Description of data used for fitting**

Number of malaria cases

The maximum and minimum monthly number of *P falciparum* cases was extracted for each year from 2004-2013 for Western and Northeastern regions from [29]. Values were extracted from figures using the WebPlotDigitizer tool [45]. To derive likelihoods, we initially assumed these cases arose from a negative binomial distribution with mu = rep (=number of reported cases from the model), and size = rep/10. The negative binomial was chosen to allow for additional uncertainty that would not be captured in a simple Poisson model.

From 2014-2019, we did not have access to similar data disaggregated by season and region. Instead, we based our likelihoods on values of annual RDT positives for the whole country extracted from [46]. For each region, we assumed the sum of the “low” and “high” monthly number of cases would be proportional to the annual number of RDT positives, and took this proportion to be the average of its values from 2012 and 2013 (as RDT use was already common by that point). We then split the sum into the low and high values based on their average relative sizes from 2004-2013. For this and other likelihoods, a small number (≤0.0001) was added to the results derived from the model (here, number of reported cases) to prevent errors during calibration for parameter sets resulting in zero malaria cases.

Prevalence of malaria

Prevalence surveys from October-November 2004 [17], November-December 2007 [18], November-December 2010 [36], and October-November 2013 [28] were used to develop malaria prevalence targets for these years.

The prevalence estimates from these surveys are derived for the entire population within malaria endemic zones, rather than those more narrowly called “high-risk” in this model. Because we assumed all cases occurred within the “high-risk” population, we set the PCR prevalence derived from the model to equal the number of people with malaria infections (asymptomatic or symptomatic) in the model divided by the population denominators described in the fixed parameter table above.

We assumed that all infectious individuals in the model are PCR positive, but not all are slide positive. Therefore, we transformed the model output (PCR prevalence) into slide prevalence for comparison with these data using the formula described in [3]. This transformation is used because only slide results were reported for the 2004, 2007, and 2010 surveys. When comparing this formula to the actual PCR data reported in 2013, in the East the model predicts 0.17% slide positivity, compared to 0.04% (95% exact CI 0.005%, 0.16%) slide positivity actually observed, and in the West the model predicts 0.04% slide positivity, compared to 0.02% (0.0006%, 0.13%) actually observed.

To derive likelihoods, we assumed a binomial distribution with n = (survey sample size)/5 and p = slide prevalence from the model, and the number positive is that observed in the survey/5. We divided the total number and number positive by 5 to allow for additional uncertainty, particularly due to heterogeneity within each region.

Resistance Data

Phenotypic mefloquine resistance was defined as late treatment failure (clinical or parasitological) to ASMQ, and similarly for phenotypic piperaquine resistance and DP. Data on Treatment Efficacy Studies (TES) from Cambodia from 2002-2019 were obtained from the World Health Organization Malaria Threats Map [47] as well as published studies [31,48–53].

Genotypic piperaquine resistance was defined as having two or more copies of *plasmepsin 2* (pfpm2). Data from 2003-2019 were obtained from published studies [32,34] and the WHO Malaria Threats Map.

Genotypic mefloquine resistance was defined as having two or more copies of the multidrug resistance protein-1 gene (*pfmdr1*). Data from 2002-2019 were obtained from published studies [30,33–35,54,55] and the WHO Malaria Threats Map.

For all of the above, the designation of whether a sample derived from a Western or Eastern province was made using the same designation as the 2010 and 2013 malaria prevalence surveys [28,36]. Western: Banteay Meanchey, Battambang, Kampong Speu, Kampot, Koh Kong, Oddar Meanchey, Pailin, Preah Vihear, Pursat, and Siem Reap. Eastern: Kampong Cham, Kampong Chhnang, Kampong Thom, Kep, Kratie, Mondulkiri, Rattanakiri, Sihanoukville, Stung Treng, and Takeo.

Binomial likelihoods were applied using the same procedure as for malaria prevalence.

**2. Model fits**

The figures below show a sample of 100 model runs taken from the posterior distribution of the model parameters (black lines). The red dots show data points that were used in defining likelihoods. Note that data points pre-2010 were not considered when fitting genotypic and phenotypic mefloquine and piperaquine resistance in Eastern Cambodia, and data points pre-2008 were not considered when fitting genotypic and phenotypic piperaquine resistance in Western Cambodia.

*Reported Monthly Cases*

Eastern Cambodia:

Western Cambodia:

*Prevalence (PCR)*

East:

West:

*Proportion of new cases with genotypic mefloquine resistance (multiple copies of pfmdr1)*

East:

West:

*Proportion of new cases with genotypic mefloquine resistance (multiple copies of pfpm2)*

East:

West:

*Proportion of new cases with phenotypic mefloquine resistance*

East:

West:

*Proportion of new cases with phenotypic piperaquine resistance*

East:

West:

*Population distribution in 2020*

Under the baseline parameter set, the average proportion of individuals in different compartment classes in 2020 was as follows:

East:

Susceptible (S): 5%

Exposed or infected, from susceptible (E or I_s): 2%

Recovered/partially immune (R): 42%

Asymptomatically infected (I_a): 52%

West:

Susceptible (S): 24%

Exposed or infected, from susceptible (E or I_s): 4%

Recovered/partially immune (R): 51%

Asymptomatically infected (I_a): 20%

**3. Extended duration of chemoprophylaxis**

The figure below shows model predictions if the average duration of chemoprophylaxis is extended to 10 years. The beneficial effects of PPQ chemoprophylaxis are lost within 1-2 years due to rapid increases in resistance.

**References**

[1] Slater HC, Ross A, Ouedraogo AL, White LJ, Nguon C, Walker PGT, et al. Assessing the impact of next-generation rapid diagnostic tests on Plasmodium falciparum malaria elimination strategies. Nature 2015;528:S94-101. https://doi.org/10.1038/nature16040.

[2] Vantaux A, Samreth R, Piv E, Khim N, Kim S, Berne L, et al. Contribution to Malaria Transmission of Symptomatic and Asymptomatic Parasite Carriers in Cambodia. J Infect Dis 2018;217:1561–8. https://doi.org/10.1093/infdis/jiy060.

[3] Okell LC, Bousema T, Griffin JT, Ouedraogo AL, Ghani AC, Drakeley CJ. Factors determining the occurrence of submicroscopic malaria infections and their relevance for control. Nat Commun 2012;3:1237. https://doi.org/10.1038/ncomms2241.

[4] Imwong M, Nguyen TN, Tripura R, Peto TJ, Lee SJ, Lwin KM, et al. The epidemiology of subclinical malaria infections in South-East Asia: findings from cross-sectional surveys in Thailand-Myanmar border areas, Cambodia, and Vietnam. Malar J 2015;14:381. https://doi.org/10.1186/s12936-015-0906-x.

[5] Scott N, Ataide R, Wilson DP, Hellard M, Price RN, Simpson JA, et al. Implications of population-level immunity for the emergence of artemisinin-resistant malaria: a mathematical model. Malar J 2018;17. https://doi.org/10.1186/s12936-018-2418-y.

[6] Aguas R, White LJ, Snow RW, Gomes MGM. Prospects for malaria eradication in sub-Saharan Africa. PloS One 2008;3:e1767. https://doi.org/10.1371/journal.pone.0001767.

[7] Hung T-Y, Davis TME, Ilett KF, Karunajeewa H, Hewitt S, Denis MB, et al. Population pharmacokinetics of piperaquine in adults and children with uncomplicated falciparum or vivax malaria. Br J Clin Pharmacol 2004;57:253–62.

[8] White NJ. How antimalarial drug resistance affects post-treatment prophylaxis. Malar J 2008;7:9. https://doi.org/10.1186/1475-2875-7-9.

[9] Sagara I, Sangare D, Dolo G, Guindo A, Sissoko M, Sogoba M, et al. A high malaria reinfection rate in children and young adults living under a low entomological inoculation rate in a periurban area of Bamako, Mali. Am J Trop Med Hyg 2002;66:310–3.

[10] Nguyen T-N, von Seidlein L, Nguyen T-V, Truong P-N, Hung SD, Pham H-T, et al. The persistence and oscillations of submicroscopic Plasmodium falciparum and Plasmodium vivax infections over time in Vietnam: an open cohort study. Lancet Infect Dis 2018;18:565–72. https://doi.org/10.1016/S1473-3099(18)30046-X.

[11] Aguas R, Maude RJ, Gomes MGM, White LJ, White NJ, Dondorp AM. Infectivity of Chronic Malaria Infections and Its Consequences for Control and Elimination. Clin Infect Dis Off Publ Infect Dis Soc Am 2018;67:295–302. https://doi.org/10.1093/cid/ciy055.

[12] Bousema T, Drakeley C. Epidemiology and infectivity of Plasmodium falciparum and Plasmodium vivax gametocytes in relation to malaria control and elimination. Clin Microbiol Rev 2011;24:377–410. https://doi.org/10.1128/CMR.00051-10.

[13] Tripura R, Peto TJ, Chalk J, Lee SJ, Sirithiranont P, Nguon C, et al. Persistent Plasmodium falciparum and Plasmodium vivax infections in a western Cambodian population: implications for prevention, treatment and elimination strategies. Malar J 2016;15:181. https://doi.org/10.1186/s12936-016-1224-7.

[14] Ittarat W, Pickard AL, Rattanasinganchan P, Wilairatana P, Looareesuwan S, Emery K, et al. Recrudescence in artesunate-treated patients with falciparum malaria is dependent on parasite burden not on parasite factors. Am J Trop Med Hyg 2003;68:147–52.

[15] Giao PT, Binh TQ, Kager PA, Long HP, Van Thang N, Van Nam N, et al. Artemisinin for treatment of uncomplicated falciparum malaria: is there a place for monotherapy? Am J Trop Med Hyg 2001;65:690–5.

[16] Diem Thuy LT, Na-Bangchang K, Hung LN, Chong MT, Van Thang N, Van Binh N, et al. Clinical efficacy of high dose monotherapy of oral dihydroartemisinin in uncomplicated falciparum malaria in viet nam. Jpn J Infect Dis 2007;60:161–6.

[17] National Institute of Public Health, Cambodia (NIPH), Malaria Consortium. Report of the Cambodia National Malaria Baseline Survey 2004. 2005.

[18] National Institute of Public Health, Cambodia (NIPH), Malaria Consortium. Cambodia Malaria Survey 2007 Report. 2007.

[19] Yeung S, Van Damme W, Socheat D, White NJ, Mills A. Access to artemisinin combination therapy for malaria in remote areas of Cambodia. Malar J 2008;7:96. https://doi.org/10.1186/1475-2875-7-96.

[20] Incardona S, Vong S, Chiv L, Lim P, Nhem S, Sem R, et al. Large-scale malaria survey in Cambodia: novel insights on species distribution and risk factors. Malar J 2007;6:37. https://doi.org/10.1186/1475-2875-6-37.

[21] Tripura R, Peto TJ, Veugen CC, Nguon C, Davoeung C, James N, et al. Submicroscopic Plasmodium prevalence in relation to malaria incidence in 20 villages in western Cambodia. Malar J 2017;16:56. https://doi.org/10.1186/s12936-017-1703-5.

[22] Mandal S, Sarkar RR, Sinha S. Mathematical models of malaria--a review. Malar J 2011;10:202. https://doi.org/10.1186/1475-2875-10-202.

[23] Baton LA, Ranford-Cartwright LC. Spreading the seeds of million-murdering death: metamorphoses of malaria in the mosquito. Trends Parasitol 2005;21:573–80. https://doi.org/10.1016/j.pt.2005.09.012.

[24] Vaughan JA. Population dynamics of Plasmodium sporogony. Trends Parasitol 2007;23:63–70. https://doi.org/10.1016/j.pt.2006.12.009.

[25] Nair S, Nash D, Sudimack D, Jaidee A, Barends M, Uhlemann A-C, et al. Recurrent gene amplification and soft selective sweeps during evolution of multidrug resistance in malaria parasites. Mol Biol Evol 2007;24:562–73. https://doi.org/10.1093/molbev/msl185.

[26] Smith DL, Drakeley CJ, Chiyaka C, Hay SI. A quantitative analysis of transmission efficiency versus intensity for malaria. Nat Commun 2010;1:108. https://doi.org/10.1038/ncomms1107.

[27] National Institute of Statistics, Ministry of Planning. General Population Census of Cambodia 2008: Provisional Population Totals. Phnom Penh, Cambodia: 2008.

[28] National Centre for Parasitology, Entomology and Malaria Control (CNM), Malaria Consortium, UN Office for Project Services. Cambodia Malaria Survey 2013. 2013.

[29] Maude RJ, Nguon C, Ly P, Bunkea T, Ngor P, Canavati de la Torre SE, et al. Spatial and temporal epidemiology of clinical malaria in Cambodia 2004-2013. Malar J 2014;13:385. https://doi.org/10.1186/1475-2875-13-385.

[30] Lim P, Alker AP, Khim N, Shah NK, Incardona S, Doung S, et al. Pfmdr1 copy number and arteminisin derivatives combination therapy failure in falciparum malaria in Cambodia. Malar J 2009;8:11. https://doi.org/10.1186/1475-2875-8-11.

[31] Denis MB, Tsuyuoka R, Poravuth Y, Narann TS, Seila S, Lim C, et al. Surveillance of the efficacy of artesunate and mefloquine combination for the treatment of uncomplicated falciparum malaria in Cambodia. Trop Med Int Health TM IH 2006;11:1360–6. https://doi.org/10.1111/j.1365-3156.2006.01690.x.

[32] Witkowski B, Duru V, Khim N, Ross LS, Saintpierre B, Beghain J, et al. A surrogate marker of piperaquine-resistant Plasmodium falciparum malaria: a phenotype-genotype association study. Lancet Infect Dis 2017;17:174–83. https://doi.org/10.1016/S1473-3099(16)30415-7.

[33] Lim P, Dek D, Try V, Sreng S, Suon S, Fairhurst RM. Decreasing pfmdr1 copy number suggests that Plasmodium falciparum in Western Cambodia is regaining in vitro susceptibility to mefloquine. Antimicrob Agents Chemother 2015;59:2934–7. https://doi.org/10.1128/AAC.05163-14.

[34] Amato R, Lim P, Miotto O, Amaratunga C, Dek D, Pearson RD, et al. Genetic markers associated with dihydroartemisinin-piperaquine failure in Plasmodium falciparum malaria in Cambodia: a genotype-phenotype association study. Lancet Infect Dis 2017;17:164–73. https://doi.org/10.1016/S1473-3099(16)30409-1.

[35] Shah NK, Alker AP, Sem R, Susanti AI, Muth S, Maguire JD, et al. Molecular surveillance for multidrug-resistant Plasmodium falciparum, Cambodia. Emerg Infect Dis 2008;14:1637–40. https://doi.org/10.3201/eid1410.080080.

[36] National Centre for Parasitology, Entomology and Malaria Control (CNM), Malaria Consortium. Cambodia Malaria Survey 2010. 2010.

[37] Yeung S, Lawford HLS, Tabernero P, Nguon C, van Wyk A, Malik N, et al. Quality of antimalarials at the epicenter of antimalarial drug resistance: results from an overt and mystery client survey in Cambodia. Am J Trop Med Hyg 2015;92:39–50. https://doi.org/10.4269/ajtmh.14-0391.

[38] Leang R, Ros S, Duong S, Navaratnam V, Lim P, Ariey F, et al. Therapeutic efficacy of fixed dose artesunate-mefloquine for the treatment of acute, uncomplicated Plasmodium falciparum malaria in Kampong Speu, Cambodia. Malar J 2013;12. https://doi.org/10.1186/1475-2875-12-343.

[39] Hansen MC, Potapov PV, Moore R, Hancher M, Turubanova SA, Tyukavina A, et al. High-resolution global maps of 21st-century forest cover change. Science 2013;342:850–3. https://doi.org/10.1126/science.1244693.

[40] World Health Organization. Artemisinin resistance and artemisinin-based combination therapy efficacy: Status report. 2018.

[41] World Health Organization. Status report on artemisinin resistance. 2014.

[42] World Health Organization. Country profile: Cambodia. n.d.

[43] Nair S, Li X, Arya GA, McDew-White M, Ferrari M, Nosten F, et al. Fitness Costs and the Rapid Spread of kelch13-C580Y Substitutions Conferring Artemisinin Resistance. Antimicrob Agents Chemother 2018;62. https://doi.org/10.1128/AAC.00605-18.

[44] Rosenthal PJ. The interplay between drug resistance and fitness in malaria parasites. Mol Microbiol 2013;89:1025–38. https://doi.org/10.1111/mmi.12349.

[45] Rohatgi A. WebPlotDigitizer 2018. https://automeris.io/WebPlotDigitizer/ (accessed October 25, 2018).

[46] World Health Organization. World Malaria Report. 2020.

[47] World Health Organization. Malaria Threats Map: Parasite drug resistance 2018. http://apps.who.int/malaria/maps/threats/ (accessed October 25, 2018).

[48] Lon C, Manning JE, Vanachayangkul P, So M, Sea D, Se Y, et al. Efficacy of two versus three-day regimens of dihydroartemisinin-piperaquine for uncomplicated malaria in military personnel in northern Cambodia: an open-label randomized trial. PloS One 2014;9:e93138. https://doi.org/10.1371/journal.pone.0093138.

[49] Amaratunga C, Lim P, Suon S, Sreng S, Mao S, Sopha C, et al. Dihydroartemisinin-piperaquine resistance in Plasmodium falciparum malaria in Cambodia: a multisite prospective cohort study. Lancet Infect Dis 2016;16:357–65. https://doi.org/10.1016/S1473-3099(15)00487-9.

[50] Leang R, Barrette A, Bouth DM, Menard D, Abdur R, Duong S, et al. Efficacy of dihydroartemisinin-piperaquine for treatment of uncomplicated Plasmodium falciparum and Plasmodium vivax in Cambodia, 2008 to 2010. Antimicrob Agents Chemother 2013;57:818–26. https://doi.org/10.1128/AAC.00686-12.

[51] Spring MD, Lin JT, Manning JE, Vanachayangkul P, Somethy S, Bun R, et al. Dihydroartemisinin-piperaquine failure associated with a triple mutant including kelch13 C580Y in Cambodia: an observational cohort study. Lancet Infect Dis 2015;15:683–91. https://doi.org/10.1016/S1473-3099(15)70049-6.

[52] Janssens B, van Herp M, Goubert L, Chan S, Uong S, Nong S, et al. A randomized open study to assess the efficacy and tolerability of dihydroartemisinin-piperaquine for the treatment of uncomplicated falciparum malaria in Cambodia. Trop Med Int Health TM IH 2007;12:251–9. https://doi.org/10.1111/j.1365-3156.2006.01786.x.

[53] Rogers WO, Sem R, Tero T, Chim P, Lim P, Muth S, et al. Failure of artesunate-mefloquine combination therapy for uncomplicated Plasmodium falciparum malaria in southern Cambodia. Malar J 2009;8:10. https://doi.org/10.1186/1475-2875-8-10.

[54] Chaorattanakawee S, Lon C, Jongsakul K, Gawee J, Sok S, Sundrakes S, et al. Ex vivo piperaquine resistance developed rapidly in Plasmodium falciparum isolates in northern Cambodia compared to Thailand. Malar J 2016;15:519. https://doi.org/10.1186/s12936-016-1569-y.

[55] Dondorp AM, Nosten F, Yi P, Das D, Phyo AP, Tarning J, et al. Artemisinin resistance in Plasmodium falciparum malaria. N Engl J Med 2009;361:455–67. https://doi.org/10.1056/NEJMoa0808859.
