# Supplementary material for: Novel anti-malarial drug strategies to prevent artemisinin partner drug resistance: A model-based analysis
Source: PLoS Comput Biol. 2021 Mar 25;17(3):e1008850. doi: 10.1371/journal.pcbi.1008850 (PMC8023453; doi:10.1371/journal.pcbi.1008850)
Supplement: Attachment — Submitted filename: response_reviewers_CLEAN.docx [file pcbi.1008850.s002.docx]

We would like to take all three reviewers for the time and effort they have spent in reviewing this manuscript. We request that all reviewers please note the updated figures, based on recommended changes to the model and added data, which do show slightly different trajectories than before. Our detailed responses to reviewers’ comments are below.

**Reviewer #1:  The emergence of drug resistance is a real threat to malaria transmission and studies that try to understand its spread are of paramount importance. This work attempts to model the potential impacts of anti-malarial drug strategies to understand artemisinin partner drug resistance using data from Cambodia. The main text is well written and ease follow as well as the model schematic shown in Figure 1.**

**However, by reading the Supporting Information Section we find many essential information about methods and data, and notice that the model presented is much more complex than what is presented in the main text. The authors performed too much data manipulation, stated too many assumptions, and designed a model with too many parameters (obtained from other articles or assumed values for 24 fixed parameters, plus 9 time-dependent parameters and fitted the model for another 8 parameters) that is difficult to not be sceptical about inferences made. Moreover, the reported monthly cases model output from Western Cambodia presents a poor fitting. The same poor fitting is observed for the prevalence (PCR) plots.**

Thank you for taking the time to review this paper.

Although the fit is imperfect, as you have noted, the aim of the model is not to quantitatively reproduce the exact past epidemiology of malaria in Cambodia, but rather to provide useful qualitative insights into the effectiveness of different anti-malarial drug strategies. In addition to simplifications made to the model to focus on these aspects, there are also limitations in the underlying data that would make it challenging for a model to fully reproduce the observed trends (such as geographic heterogeneities beyond those captured here and variable underreporting of malaria cases over time).

Nevertheless, the model does capture, at least in part, many of the significant changes in malaria policy in Cambodia over the past 20 years. These include repeated changes in first-line treatment; increasing availability of first-line treatment, with greater crackdowns on unauthorized or counterfeit drugs; and declining malaria risk due to deforestation and/or greater ITN coverage.

We believe that the fact that the model predicts similar qualitative trends in two (separately fit) regions of Cambodia, and across a range of sensitivity analyses, to be a reassuring sign of the robustness of the model. Additionally, the model provides interesting insights into the dynamics of piperaquine and mefloquine resistance in this region, despite limitations in its quantitative predictions.

**Reviewer #2: The authors have conducted a no trivial work aiming to contain the spread of artemisinin’s partners drugs resistance. Their final goal seems to prevent the spread of artemisinin’s resistance outside the GMS to Africa by preventing its partner drug resistance.** **The authors use a simple compartmental seir model for malaria transmission to assess the capacity of 3 scenarios (current “ASMQ treatment”, triple-ACT “DP+MQ” and treatment + prophylaxis “ASMQ+DP”) to contain the partner drug resistance. The modeling framework seems appropriate with fair assumptions. The paper is well written but should be improved.**

**Major comments**

**1) Baseline results
Please add 2018-2020 incidence / resistance data if any to figure 2.**

As this comment notes, we originally had very few data points from 2018-2020. In response to your suggestion, we searched for more recent data on incidence and resistance. We were able to find new data through 2019 on both incidence and resistance, compared to when the paper was first submitted. We have incorporated these new data into the likelihoods and re-fit the model (after additionally allowing some symptomatic individuals to move direct to asymptomatic, following the recommendation of Reviewer 3). We also added these new data to the plots in Section 2 of the Appendix, and a reference to this section in the main text. Overall the model fits to the new data appear reasonable, except that there appears to be a short (6 month-1 year) delay between the fall in piperaquine resistance observed in the data and that predicted by the model. This could suggest that the dates of the transition from DP to ASMQ as first-line treatment in the model are slightly off. Indeed, we have input these as fixed dates into the model, whereas in reality the transition was not instantaneous.

Note that the reference for malaria incidence post-2013 has changed to allow for inclusion of these new data. However, the trends are still similar to what was presented before.

**2) According to the authors, Cambodia has several times switched between ASMQ and DP and currently back to ASMQ. The authors believe ASMQ efficacy could be short-lived triggering their work to support novel treatment strategies. Since ASMQ and DP have inconsistent, why not test/suggest new drugs? The authors alluded for instance to AL as a possible candidate and I wonder why ASMQ and DP remain their preferred drugs in their suggested treatment scenarios. I am just trying to get more background / literature regarding the choice of treatment strategies / drugs by the authors.**

Thank you for this question. AL could be another possible candidate for use in Cambodia and the rest of the Greater Mekong Subregion as either treatment or chemoprophylaxis. However, there are some concerns about its use. First, lumefantrine is widely used across Africa, and the impacts of resistance to this partner drug developing in Cambodia (a known hotbed of resistance) and spreading to Africa could be severe. Second, lumefantrine has a relatively short half-life (compared to piperaquine and mefloquine) and requires multiple doses per day, which raises some concerns about compliance, as well as appropriateness for prevention. Low-grade lumefantrine resistance in the region has also been noted. Pyronaridine-artesunate is another drug combination that has been discussed as an option for both treatment or chemoprophylaxis. However, it has generally been considered inappropriate as chemoprophylaxis based on concerns about safety, and the fact that pyronaridine may represent a drug of last resort in the Greater Mekong Subregion [1].

For these reasons, a solution focused on the two partner drugs already used historically in Cambodia, piperaquine and mefloquine, would be ideal and was the focus of this paper. Making model predictions about resistance trends in resistance to lumefantrine and pyronaridine would also be more difficult, as these drugs have not yet been widely used in Cambodia, and it would be difficult to choose appropriate fitness parameters in the absence of sufficient data from the region.

To clarify these points, we have added the following text to the Discussion: “However, these alternative ACTs also have major drawbacks. Lumefantrine has a relatively short half-life and more complicated dosing schedule compared to piperaquine or mefloquine; it is also the primary partner drug used in Africa, which would heighten concerns about resistance. The safety of repeated courses of pyronaridine is currently unknown, and it represents a possible drug of last resort without pre-existing resistance in the GMS.” (Line 275).

**3) Cambodia is not a high burden country; the urgency remains elimination rather than changing policy to reduce incidence. Why is prophylaxis important here? Maintaining ASMQ+DP in the paper does not add any value to the purpose of this work since rather than preventing partner drugs’ resistance, the prophylaxis is increasing the spread of such resistance. To me the prophylaxis's strategy simply adds more complexity and distraction to the paper with no benefit that all. Since the main purpose of their work is to prevent resistance, the authors could for instance test and suggest a treatment/prophylaxis strategy that reduces both incidence and resistance, or simply leave it out if not effective, or please justify why it is so crucial to this paper.**

As noted, the urgency in Cambodia is malaria elimination. Malaria in Cambodia can be considered an occupational disease of certain high-risk groups who travel to the forest for their livelihoods [2,3]. Stopping infections in these individuals is now high priority to achieve elimination. As a result, multiple studies are currently being conducted in Cambodia employing various forms of chemoprophylaxis to high-risk forest-goers. Furthermore, the provision of “intermittent preventive treatment to travelers who visit high-risk areas” (another name for chemoprophylaxis among forest-goers) is now considered part of the official national strategy to eliminate Cambodia (<https://www.who.int/cambodia/news/detail/03-11-2020-cambodia-commits-to-eliminating-plasmodium-falciparum-malaria>). We feel it is important to consider the chemoprophylaxis strategy, given its relevance to current plans for malaria elimination in Cambodia.

**4) I have a problem with the “Rate of waning protection by treatment drug”. The parameter (w_t) value seems generic and attributed to all drugs. Artemisinin and partner drugs have different half-lives and for that reason, a clarification should be given to how PKPD model parameters were fitted to drugs' half-lives. If there are assumptions on half-lives, it should be clearly stated in the text but it can't be 20days for artemisinin and partner drugs. Additionally, this (drug kinetics) may be useful to sensitivity analysis to assess differential spreads of resistance between treatment strategies but I leave it to the authors to decide whether they want to pursue and exploration.**

To clarify this point, we have renamed this parameter “rate of waning protection by artemisinin partner drug” (Table entry starting “w_t”, table starting Line 15, Appendix). Artemisinin has a short half-life compared to the partner drugs and the long-term protection provided by ACTs comes primarily from the partner drug. Estimates of the half-lives of mefloquine and piperaquine are largely similar, at around 15-30 days for mefloquine and 20-35 days for piperaquine [4,5].

**5) The discussion section will benefit from a paragraph on safety / toxicity of triple-ACT or combination of treatment / prophylaxis, and possibly a sentence on cost-effectiveness.**

We have added the following text to the Discussion (line 286).

Safety is another consideration when combining or repeating antimalarial use, as in the scenarios here. A clinical trial of DP-MQ found that the rate of clinical adverse events was not significantly increased compared to DP. Furthermore, the observed increase in QT interval with DP-MQ was not greater than that with DP [6]. Safety concerns are amplified when an antimalarial is given as chemoprophylaxis, due to its repeated and more widespread use in apparently healthy individuals. Existing data support the safety of monthly DP as intermittent preventive treatment or chemoprophylaxis [7]. Cost-effectiveness is another aspect that could be considered, as well as the infrastructure needed to implement each intervention (ex. could existing village malaria workers dispense monthly chemoprophylaxis, or is another system needed?)

**Minor
1) Would be more informative to the reader to have some numbers / statistics about artemisinin resistance and its partner drugs resistance in the GMS, added to the background/introduction.**

We have added the following sentence to the background (line 75).

A study conducted from 2015-2018 in Thailand, Vietnam, and Cambodia found the efficacy of dihydroartemisinin-piperaquine (DP) at day 42 was only 50%, and over 90% of patients’ samples showed mutations in the kelch13 gene associated with artemisinin resistance.

**2) Fig 1a is not cited in the text.**

We have moved the following sentence to the Methods subsection on modeling partner drug resistance (line 392).

Figure 1 shows a subset of the model states: those pertaining to drug susceptible infections (Fig 1a) and those pertaining to genotypically PPQ-resistant infections (Fig 1b).

**Reviewer #3: In this study the authors develop a model, parameterised to reflect malaria transmission in Cambodia, to explore the impact of triple artemisinin combination therapies (ACTs) and chemoprophylaxis in limiting spread of drug-resistant parasites and accelerate elimination. This is an interesting subject to explore in detail. The complexity of the interplay between drug-sensitive & drug resistant parasites has been simplified in the model, but I think it has been done in an appropriate way, given the aims of the study. The results obtained in the baseline model, that triple ACT therapy could reverse mefloquine resistance, but not impact overall incidence, and the chemoprophylaxis did impact incidence but encouraged the spread of drug-resistant parasites, are intuitive but important to explore in detail. Furthermore, the sensitivity analysis, which explores the changes in model assumptions required for triple ACT treatment to promote spread of parasites resistant to both mefloquine and piperaquine is very useful. I have some minor comments that I would like to be addressed, and I am happy to recommend the work for publication once this has been done.**


**1) Lines 103-105. The authors state that partner drug resistance more important in driving treatment failure than artemisinin resistance. Would they be able to provide a reference for this?**

We have rephrased this statement to soften the language slightly: “We focus on partner drug resistance, rather than resistance to artemisinin, as artemisinin resistance is already widespread in Cambodia and, on its own, rarely leads to treatment failure.” (Line 106). We have also added two references.

**2) Line 146: Define DS. Drug susceptible?**

Yes; we have added this definition. (Line 351)

**3) Regarding treatment coverage: parameter ‘app’ governs the proportion of symptomatic individuals receiving first line treatment. What proportion of symptomatic individuals receive non-recommended treatment (e.g. monotherapy)? If it is (1-app), is it realistic to assume that 100% of symptomatic infections are treated? If not, what would the implications be for the modelling results presented here?**

We had assumed that all individuals would receive some form of treatment, and thus (1-app) would be individuals receiving monotherapy or other treatment. As we assume that 50% of those treated with inappropriate therapy recrudesce, this category could in theory also include those who were not treated with any sort of anti-malarial drug. We have added “or individuals receiving no treatment” to the supplement table entry for s_a.

Previously, we had assumed simply maintained all those with recrudescence in the “symptomatic infectious” compartment. However, in response to your next comment, we now allow 10% of these to transition to the “asymptomatic infectious” compartment.

**4) Regarding Figure 1, the upper schematic implies that state nIa (asymptomatic, infectious individuals) can only be reached through the recovered state, R. Presumably this is incorrect?**

This was the initial assumption of our model – admittedly an oversimplification – that immunologically naïve infected individuals remained in the symptomatic infectious compartment until recovered. In reality, of course, it is possible some of these individuals may cycle back and forth between symptomatic and asymptomatic over a longer period of time. We have now modified the model to allow 10% of those who fail treatment (including due to inappropriate or no treatment – see note above) to move directly into the asymptomatic infectious category. (We also experimented with higher proportions, but found that the model was then no longer able to replicate the observed fall in piperaquine resistance post-2017).

We have now:

1) Added arrows for this new transition to Figure 1.

2) Added a new row to the fixed parameter table in the Appendix (table beginning line 15, line below s_p, Appendix).

**5) Figure 2 legend: I’m not sure called treatment with a triple ACT “Joint Treatment” is sensible, but I leave this choice to the authors.**

We have replaced the phrase “joint treatment” with “triple ACT treatment” throughout the main text. We have also changed it in Figures 2-5.

**6) The authors demonstrate that the chemoprophylaxis scenario decreased malaria incidence, but the policy is only explored for 1 year. It would be interesting to see what happens if the policy is pursued e.g. do these decreases continue, or does the increased prevalence of drug-resistant malaria eventually render the intervention ineffective?**

We have re-run the code assuming an average duration of prophylaxis of 10 years. The results are shown in the Appendix Section 3 and reference in the main text (line 167). Even with this longer duration, the beneficial effects of chemoprophylaxis are lost within 1-2 years, apparently because of the rapid increases in PPQ resistance.

**Editor: Please provide the code in a more suitable manner.**

The code is now available at the following GitHub repository: https://github.com/agkunkel/cambodia-partner-drugs

**References**

[1]	von Seidlein L, Peto TJ, Tripura R, Pell C, Yeung S, Kindermans JM, et al. Novel Approaches to Control Malaria in Forested Areas of Southeast Asia. Trends Parasitol 2019;35:388–98. https://doi.org/10.1016/j.pt.2019.03.011.

[2]	Bannister-Tyrrell M, Gryseels C, Sokha S, Dara L, Sereiboth N, James N, et al. Forest Goers and Multidrug-Resistant Malaria in Cambodia: An Ethnographic Study. Am J Trop Med Hyg 2019;100:1170–8. https://doi.org/10.4269/ajtmh.18-0662.

[3]	Sanann N, Peto TJ, Tripura R, Callery JJ, Nguon C, Bui TM, et al. Forest work and its implications for malaria elimination: a qualitative study. Malar J 2019;18:376. https://doi.org/10.1186/s12936-019-3008-3.

[4]	Karbwang J, White NJ. Clinical pharmacokinetics of mefloquine. Clin Pharmacokinet 1990;19:264–79. https://doi.org/10.2165/00003088-199019040-00002.

[5]	Thanh NX, Trung TN, Phong NC, Quang HH, Dai B, Shanks GD, et al. The efficacy and tolerability of artemisinin-piperaquine (Artequick®) versus artesunate-amodiaquine (Coarsucam^TM^) for the treatment of uncomplicated Plasmodium falciparum malaria in south-central Vietnam. Malar J 2012;11:217. https://doi.org/10.1186/1475-2875-11-217.

[6]	van der Pluijm RW, Tripura R, Hoglund RM, Pyae Phyo A, Lek D, Ul Islam A, et al. Triple artemisinin-based combination therapies versus artemisinin-based combination therapies for uncomplicated Plasmodium falciparum malaria: a multicentre, open-label, randomised clinical trial. Lancet Lond Engl 2020;395:1345–60. https://doi.org/10.1016/S0140-6736(20)30552-3.

[7]	Gutman J, Kovacs S, Dorsey G, Stergachis A, Ter Kuile FO. Safety, tolerability, and efficacy of repeated doses of dihydroartemisinin-piperaquine for prevention and treatment of malaria: a systematic review and meta-analysis. Lancet Infect Dis 2017;17:184–93. https://doi.org/10.1016/S1473-3099(16)30378-4.
