# Supplementary material for: Novel anti-malarial drug strategies to prevent artemisinin partner drug resistance: A model-based analysis
Source: PLoS Comput Biol. 2021 Mar 25;17(3):e1008850. doi: 10.1371/journal.pcbi.1008850 (PMC8023453; doi:10.1371/journal.pcbi.1008850)
Supplement: Attachment — Submitted filename: response_reviewers_2.docx [file pcbi.1008850.s003.docx]

We would like to thank Reviewer #1 for these additional opportunities to improve our manuscript. Detailed responses are available below.

**The work attempts to model the potential impacts of anti-malarial drug strategies to understand artemisinin partner drug resistance using data from Cambodia. Surprisingly, the overall malaria incidence trend presented in Figures 2-5 does not change over time and across different scenarios. If we look at Figure 2, for example, under the Status Quo scenario, the model output indicates that the number of cases per month in West Cambodia does not increase when the proportion of new infections with multiple copy number pfmdr1 goes from around 0.1 in 2018 to around 0.9 in 2025. Figures 3 and 4 bring similar results. If we take Figure 5, even when the proportion of new infections with multiple copy number pfmdr1, pfpm2 or pfmdr1+pfpm2, is close to 1 (panels D, F and H), the overall malaria incidence predicted by the model also does not increase over the time. As mentioned on the text, if all innervations are held constant and those with late treatment failure re-enter the I_s compartment immediately, why the spread of artemisinin partner drug resistance does not affect the overall malaria?**

Thank you for this question. The fact that we do not see an appreciable increase in malaria incidence despite spread of multiple copies of pfmdr1 can be attributed to the following factors. First, there is a fitness cost associated with carrying this genotype. Second, the impact on treatment failure is relatively limited. We assume that 75% of individuals treated with ASMQ will still be successfully treated without relapse despite carrying this genotype. Those that are not, if they re-enter the I_s compartment immediately, again have 75% chance of being successfully treated with ASMQ.

We have added the following sentence to address this (line 167): The similar trends in incidence despite differing resistance patterns likely reflect the fitness costs of resistance and the low assumed probability of phenotypic drug resistance given genotypic resistance markers.

**As we can find in Figure 1, individuals move to compartment R after recovering and become partially immune to malaria. Based on a strong assumption, the model assumes that all partially immune individuals (from compartment R) who develop malaria are asymptomatic (lines 361-362) and move to compartment I_a. After recovering from the asymptomatic infection, individuals move back to compartment R (recovered and partially immune to malaria). The rate of waning immunity (parameter “w”) is w=1/365 day^(-1), that is, after recovering from an infection (symptomatic or asymptomatic), it takes on average 365 days before any individual becoming “fully” susceptible again (compartment S). Considering that the model assumes a single high-risk population, I wonder if the rate to move from R to I_a is not considerably higher than the rate to move from R to S (waning immunity). In affirmative case, it is expected that, under model assumptions, most individuals will be “trapped” between compartments R and I_a. As a consequence, the overall malaria incidence (I am assuming only symptomatic cases) might be sensible to variations on the rate of waning immunity. In order to clarify this point, it would be interesting to have a plot of proportion of individuals in compartments S, I_s, I_a and R over time. I also wonder if the authors do not consider to perform a sensitivity analysis of the rate of waning immunity (parameter “w”). Please, clarify this point.**

To address this question, we looked at the proportion of individuals in the different compartments in 2020 under the baseline scenario. The results were as follows, and have been added to the Appendix (line 218). Given the substantial differences in the proportion of individuals in the susceptible compartment in the models parameterized to Eastern and Western Cambodia, we did not conduct additional sensitivity analyses.

*Population distribution in 2020*

Under the baseline parameter set, the average proportion of individuals in different compartment classes in 2020 was as follows:

East:

Susceptible (S): 5%

Exposed or infected, from susceptible (E or I_s): 2%

Recovered/partially immune (R): 42%

Asymptomatically infected (I_a): 52%

West:

Susceptible (S): 24%

Exposed or infected, from susceptible (E or I_s): 4%

Recovered/partially immune (R): 51%

Asymptomatically infected (I_a): 20%

**Overall, the authors concluded that triple ACTs may be useful at limiting spread of resistance to artemisinin partner drugs (lines 321-322). Although pfpm2 and pfpm2+pfmdr1 results from Figure 2 are pretty much the same for triple ACT and Status Quo regimes, the first performs better for pfmdr1. However, if my understanding is correct, sensitivity analysis results raise questions about the model robustness with respect to the conclusion and do not clearly favour triple ACT regime. We can note in Figure 3 for West Cambodia that triple ACT regime performs worse than the Status Quo regime for pfpm2 and pfpm2+pfmdr1. For a long-term analysis, the same behaviour can be found in Figure 4. Further, the authors say: “In sensitivity analyses, it was possible but difficult to create a situation under which the triple ACT scenario increased joint MQ/PPQ resistance substantially beyond its current levels” (lines 239-241). Regarding joint MQ/PPQ resistance, it seems to me that results from Figures 2-5 suggests that, overall, the Status Quo scenario performs better than the triple ACT. Please, clarify this point.**

Thank you for raising this point. The sensitivity analyses included here are all fairly extreme scenarios, intended to see the behavior of the triple ACT under unfavorable parameter sets. Nevertheless, we agree that our language may have been too positive towards the triple ACT scenario, and have modified it in the following locations.

Abstract (lines 31-40):

**Results**: We predicted MQ resistance to increase under the status quo scenario. Triple ACT treatment reversed the spread of MQ resistance, but had no impact on overall malaria incidence. Joint MQ-PPQ resistance declined under the status quo scenario for the baseline parameter set and most sensitivity analyses. Compared to the status quo, triple ACT treatment limited spread of MQ resistance but also slowed declines in PPQ resistance in some sensitivity analyses…

**Conclusions**: Triple ACTs may limit spread of MQ resistance in the Cambodia, but would have limited impact on malaria incidence and might slow declines in PPQ resistance.

Author summary (lines 67-72):

The triple ACT scenario limited the spread of mefloquine resistance but had minimal impact on the number of malaria cases. In some sensitivity analyses, it also slowed declines in piperaquine resistance. Chemoprophylaxis reduced the number of malaria cases and increased resistance, but both of those effects were short-lived. We conclude that triple ACTs may prevent the spread of partner drug resistance, but could be less effective against pre-existing resistance in the population. Additionally, triple ACTs would need to be coupled with other interventions to decrease cases.

Discussion (line 357-358):

Overall, we conclude that triple ACTs may be useful at limiting spread of resistance to artemisinin partner drugs in high risk areas like the GMS. However, they could also slow declines in pre-existing resistance compared to single use of another drug.

**Posterior distribution bounds of parameters “beta_min_m”, “p” and “f_p” are out of prior distribution bounds (Supporting Information, line 87). Please, clarify this point.**

The “upper” and “lower” columns do not represent strict bounds but rather the upper and lower ends of a 95% interval. The distributions are continuous and values outside of these ranges may be selected if supported by the data. We have clarified this in the table header (Appendix line 84)
